# Early Onset of TNFα-Driven Arthritis, Auto-inflammation, and Progressive Loss of Vision in a Patient with *ALPK1* Mutation

**DOI:** 10.1007/s10875-022-01214-8

**Published:** 2022-03-02

**Authors:** Julia Hecker, Marilena Letizia, Britt-Sabina Loescher, Britta Siegmund, Carl Weidinger

**Affiliations:** 1 Medizinische Klinik Für Gastroenterologie, Infektiologie Und Rheumatologie, Charité – Universitätsmedizin Berlin, Campus Benjamin Franklin, corporate member of Freie Universität Berlin and Humboldt-Universität Zu Berlin, Hindenburgdamm 30, 12200 Berlin, Germany; 2 Institute of Clinical Molecular Biology, Christian-Albrechts-University of Kiel, Kiel, Germany; 3 BIH Charité Clinician Scientist Program, Berlin Institute of Health, Berlin, Germany

To the Editor,

With great interest, we have read the research letter by Jamilloux et al. recently reporting three cases of patients with auto-inflammation and concomitant ectodermal dysplasia and progressive loss of vision due to mutations in the *α-kinase 1* (*ALPK1*) gene [1].

We here describe the case of a 47-year-old Caucasian female presenting herself in our outpatient clinic with a history of therapy-refractory, early-onset arthritis, progressive loss of vision, splenomegaly, leukopenia, anemia, chronic pain syndrome, disseminated atopic dermatitis, and anhidrosis. The clinical course of the patient is graphically summarized in Fig. 1A. Briefly, the patient first developed progressive loss of visual acuity at the age of 11 due to bilateral chronic uveitis posterior, inflammatory retinitis, and optic nerve edema and shortly after displayed signs of destructive arthritis starting at the proximal interphalangeal joints subsequently progressing to metacarpophalangeal joints and knees. The patient tested negative for infections, known immunodeficiencies, autoantibodies, and the HLA-B27 antigen. Initial immunosuppressive treatment consisted of methotrexate, which was discontinued when the patient developed a benign bladder tumor at the age of 17. The patient was subsequently treated for several years with sulfasalazine, under which the loss of vision gradually deteriorated. Numerous treatment approaches were subsequently initiated including rituximab, baricitinib, tofacitinib, and ustekinumab as well as inter-current applications of corticosteroids during flairs. Treatment with adalimumab was induced but discontinued briefly after due to the development of recurrent infections (Table S3 summarizes the duration of the single therapies and lists the reasons for discontinuation).

**Fig. 1:**
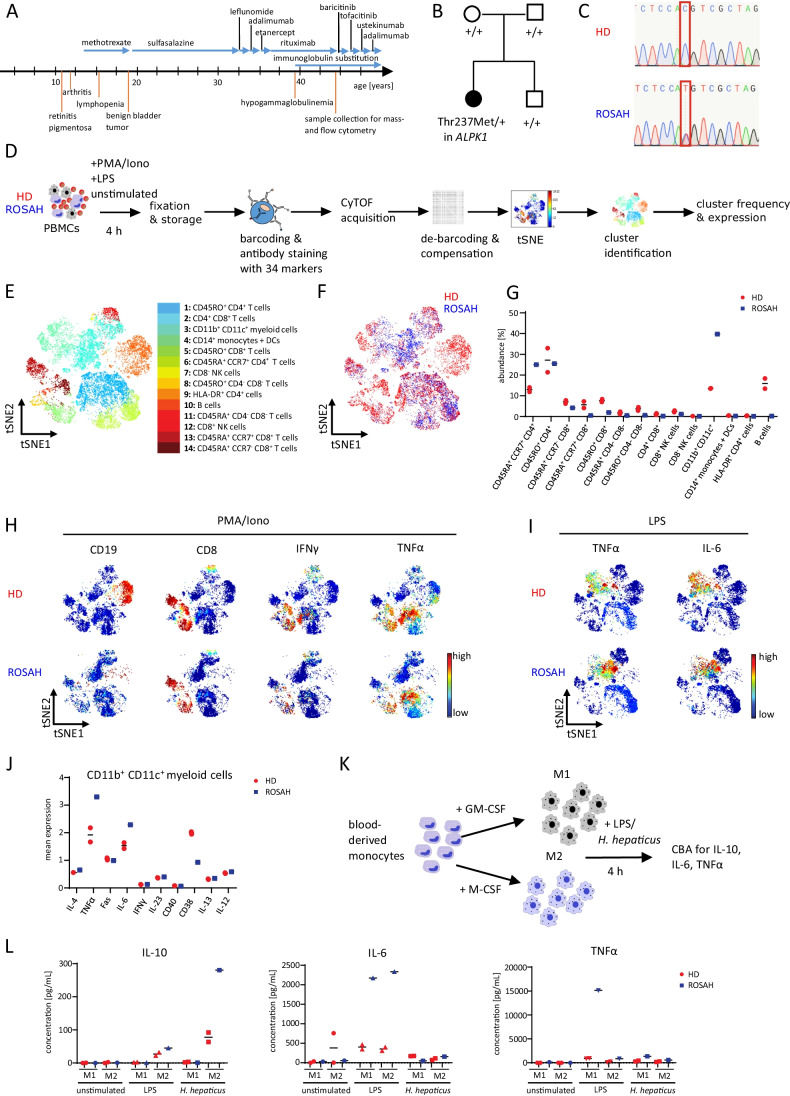
Deep immune phenotyping of a patient with chronic auto-inflammation and progressive vision loss reveals an increased expression of TNFα and IL-6 in myeloid cells. **A** Summary of the clinical history of the ROSAH patient. **B** Variant status in the *ALKP1* gene in the ROSAH patient, her parents, and her brother as assessed by whole-exome sequencing, “ + ” indicating the *wild type* allele, Thr237Met indicating the mutated allele with missense mutation (*p.Thr237Met*). **C** Sanger sequencing of EDTA blood of the ROSAH patient and an unrelated healthy donor (HD). The red square indicates the position of the mutation (c.710C) in the *ALPK1* gene. **D** Schematic summary of immune cell characterization of the ROSAH patient and two unrelated HDs by mass cytometry. Peripheral blood mononuclear cells (PBMCs) of the ROSAH patient and HDs were ex vivo stimulated with phorbol 12-myristate 13-acetate (PMA)/ionomycin (Iono) or lipopolysaccharide (LPS) for 4 h followed by fixation and mass cytometry staining and acquisition. **E** t-SNE plot of concatenated FCS files from all samples, colored by the 14 clusters identified in CD45^+^ cells. **F** t-SNE plot displaying the cellular distribution of CD45^+^ cells of the ROSAH sample (blue) and two HD samples (red). **G** Abundance of the 14 different cell clusters in PMA/Iono-stimulated PBMCs. **H** t-SNE plots of PMA/Iono-stimulated PBMCs colored by the expression of selected markers. Red represents high expression; blue represents low expression. **I** t-SNE plots of LPS-stimulated PBMCs colored by the expression of selected markers. Red represents high expression; blue represents low expression. **J** Mean expression of selected markers in CD11b^+^CD11c^+^ myeloid cells of LPS-stimulated PBMCs. **K** Blood-derived monocytes of the ROSAH patient and one HD were differentiated into M1 or M2 macrophages by supplementation of GM-CSF or M-CSF for 7 days and subsequently stimulated with LPS or *Helicobacter hepaticus* (*H. hepaticus*). **L** Concentrations of IL-10, TNFα, and IL-6 in the supernatant of stimulated macrophages. Duplicates represent two wells of separately differentiated macrophages from the same HD

Suspecting a genetic syndrome, we performed whole-exome sequencing of peripheral blood mononuclear cells (PBMCs) from the patient as well as her un-affected parents and brother and detected a de novo heterozygous missense mutation (c.710C > T, [p.Thr237Met]) in the *ALPK1* gene. This mutation was subsequently confirmed by targeted Sanger sequencing (Fig. 1B, C). Remarkably, this *ALPK1* p.Thr237Met mutation has previously been described to cause retinal dystrophy, optic nerve edema, splenomegaly, anhidrosis, and migraine headache (ROSAH) [2] and is furthermore detectable in patients with *ALPK1*-associated auto-inflammatory disorders and concomitant ectodermal dysplasia reported by Jamilloux et al. [1]. Based on these findings, we concluded that our *ALPK1*-mutated patient is suffering from ROSAH syndrome with combined early-onset arthritis and systemic auto-inflammation.

Since *ALPK1* has been described to act as an important mediator of NF-κB activation in response to Gram-negative bacteria [3], we hypothesized that the identified *ALPK1* mutation might affect immune cell function, thereby facilitating the development of systemic auto-inflammation.

We therefore compared the immune cell composition and activation of PBMCs obtained from our *ALPK1*-mutated patient to PBMCs of healthy donors (HDs) by applying mass cytometry (Fig. 1D). Detailed descriptions of antibody panels, experimental settings, and data analyses are available in the Supplementary Information. Of note, the patient received tofacitinib (5 mg bid) at the time of mass cytometric analysis of immune cells, which is very likely to have at least partially affected CyTOF results.

As shown in Fig. 1F, we observed an almost complete absence of CD19^+^ B cells in our *ALPK1*-mutated ROSAH patient when compared to HDs, which we mainly attributed to the previous treatment with rituximab two years prior to the current evaluation. However, the observation that our patient had featured decreased levels of immunoglobulins before rituximab treatment (Suppl. Figure 1) together with the sustained reduction of B cells two years after rituximab treatment could also indicate that ALPK1 plays a role in B cell homeostasis. Yet, it would be important to further validate our results in additional ROSAH patients to understand if *ALPK* is involved in the regulation of B cell homeostasis or whether the observed defects in B cells were caused pharmacologically by one of the multiple pre-treatments of our patient (Table S3).

We furthermore detected a pronounced reduction in CD8^+^ T cells as both naïve (CD45RA^+^CCR7^+^) and activated (CD45RO^+^) CD8^+^ T cells were decreased (Fig. 1F, G) and produced less IFNγ and TNFα when compared to CD8^+^ T cells of HDs (Fig. 1H, Suppl. Figure 2). To independently validate these findings, we analyzed T cells of the ROSAH patient and additional five HDs by flow cytometry, revealing similar reductions in CD8^+^ T cell frequency and expression of IFNγ and TNFα in T cells of the ROSAH patient (Suppl. Figure 3). Of note, tofacitinib treatment has been shown to reduce T cell proliferation and pro-inflammatory cytokine production [4], and we therefore cannot exclude an influence of tofacitinib treatment on T cell expansion in the ROSAH patient. However, the production of pro-inflammatory cytokines of ex vivo activated T cells obtained from patients treated with tofacitinib is only slightly reduced [4], arguing that the pronounced reduction of IFNγ and TNFα expression in T cells of the ROSAH patient might not be caused by tofacitinib treatment alone.

Thus, our data together with the reduction of lymphocytes documented in our patient’s record even before immune suppressive therapy with methotrexate, sulfasalazine, rituximab, baricitinib, tofacitinib, and ustekinumab was induced (Table S4) suggest in our opinion that *ALPK1* might at least partially control the homeostasis of lymphocytes.

In contrast, the frequency of CD11b^+^CD11c^+^ myeloid cells was increased in the ROSAH patient (Fig. 1F, G), and myeloid cells expressed augmented levels of IL-6 and TNFa upon ex vivo stimulation with lipopolysaccharide (LPS), when compared to HDs (Fig. 1I, J), highlighting that the *ALPK1* p.Thr237Met mutation might trigger auto-inflammation in a TNFα- and IL-6-dependent manner. To better characterize the myeloid compartment, blood-derived monocytes of the ROSAH patient and one HD were isolated and subsequently differentiated into either M1 or M2 macrophages via addition of GM-CSF or M-CSF for seven days (Fig. 1K). Of note, no differences were detected in the polarization of macrophages when comparing the ROSAH patient to a HD by flow cytometry or microscopy (Suppl. Figure 4), suggesting that the *ALPK1* p.Thr237Met mutation does not primarily affect the survival and differentiation of macrophages in vitro. However, when we challenged M1- or M2-like macrophages with either LPS or *Helicobacter hepaticus*, macrophages derived from the ROSAH patient produced more IL-6 and TNFα after LPS stimulation when compared to macrophages from a HD (Fig. 1L). These results further support our ex vivo findings obtained by mass cytometry and suggest an activation of NF-κB signaling in myeloid cells of the ROSAH patient, possibly caused by a gain of function of the mutated *ALPK1* as proposed by Jamilloux et al. and others [1, 2].

Based on the observed higher production of TNFα and IL-6 in *ALPK1 p.Thr237Met–*bearing macrophages and the lack of efficiency of the JAK inhibitors tofacitinib and baricitinib in our ROSAH patient, we considered the non-JAK/STAT regulated cytokine TNFα to be a key player for the development of auto-inflammatory symptoms in our ROSAH patient. Therefore, we decided to re-initiate anti-TNFα therapy with adalimumab and continued low-dose corticosteroid treatment and COX-2 inhibition with etoricoxib. This combination of immune suppression resulted in an improvement with regard to arthritis as joint swelling and pain decreased after application of adalimumab, suggesting that the integration of TNFα blockade together with corticosteroids should be primarily considered for immune suppression in patients with *ALPK1-*associated autoimmune disorders. Accordingly, a literature review of Jamilloux et al. of 21 reported patients with *APLK1*-associated diseases [1] revealed a resolution of auto-inflammation-mediated symptoms in two out of four patients treated with TNFα blockers. Interestingly, the authors of the study reported that the progression of vision loss could not be stopped by TNFα blockade [5], suggesting that the ophthalmologic symptoms of the ROSAH syndrome are rather caused by other mechanisms, possibly by cell-intrinsic functions of ALPK1 within the cilia as proposed by Williams et al. [2]. Of note, we could not assess any effect of the TNFα blockade on the ophthalmologic symptoms, as our patient was almost completely blind when adalimumab therapy was initiated.

Our data indicate that ALPK1 might play a crucial role in the function of pro-inflammatory lymphocytes and future studies will be required to substantiate the mechanisms by which the *ALPK1 p.Thr237Met* mutation confers to auto-inflammation in ROSAH patients and by which *ALPK1* controls the production of TNFα and IL-6 in macrophages. The observation that 10 of 21 reported patients with *ALPK1*-associated disease display cytopenia [1] furthermore suggests that *ALPK1* might be an important regulator of the differentiation and/or survival of hematopoietic cell lineages. Therefore, we share the opinion of Jamilloux et al. that *ALPK1* mutations should be considered in the diagnosis of patients with suspected auto-inflammatory disorders and inborn errors of immunity [1].

## Supplementary Information

Below is the link to the electronic supplementary material.

Supplementary file1 (PDF 6151 KB)

## Data Availability

The datasets used and/or analyzed during the current study are available from the corresponding authors on reasonable request that does not include confidential patient information.

## Code Availability

Code is available from the corresponding authors on reasonable request.
