# Supplementary material for: Early Onset of TNFα-Driven Arthritis, Auto-inflammation, and Progressive Loss of Vision in a Patient with ALPK1 Mutation
Source: J Clin Immunol. 2022 Mar 2;42(4):880–4. doi: 10.1007/s10875-022-01214-8 (PMC9166835; doi:10.1007/s10875-022-01214-8)
Supplement: Supplementary file 1 — Supplementary file1 (PDF 6151 KB) [file 10875_2022_1214_MOESM1_ESM.pdf]

Supplementary Information

Early onset of TNFα- driven arthritis, auto-inflammation and progressive loss of vision in a patient with *ALPK1* mutation

Hecker et al.

Patient consent and sample acquisition

Informed consent for publication of clinical details and results acquired during diagnosis (including in-vitro experiments, mass cytometry and genetic analysis) was obtained from the patient. Results shown were derived from our extended diagnostic panel used for the clinical assessment of patients with suspected, systemic immune-related disorders which includes whole exome sequencing, immune deep phenotyping by mass cytometry as well as functional in-vitro assays.

Isolation of peripheral blood mononuclear cells

Peripheral blood mononuclear cells (PBMCs) of the ROSAH patient and two healthy controls were isolated by density-gradient centrifugation using SepMate^{TM} PBMC Isolation tubes (Stemcell) and Ficoll-Paque™ PLUS (GE Healthcare) following the manufacturer's protocols. PBMCs were either used freshly or were frozen in fetal calf serum (FCS) supplemented with 10% dimethyl sulfoxide (DMSO) (Sigma-Aldrich).

Whole exome sequencing

Whole exome sequencing was performed as previously described (1). DNA was isolated from EDTA blood of the ROSAH patient, her brother and her parents. Exome enrichment was performed using the IDT xGen Exome Research Panel v 1.0 and 2x75bp paired-end sequencing was carried out on an Illumina HiSeq 3000 sequencer. The reads were subsequently mapped against the human reference genome, converted to bam format and indexed with Samtools. PCR duplicates were removed, local realignment around InDels and base quality score recalibration were performed. Subsequently, variant calling and variant quality score recalibration was performed. Variant annotation and filtering were performed using the Alissa Interpret software (Agilent).

Sanger sequencing

The *ALPK1* mutation detected by *whole exome sequencing* was subsequently confirmed by Sanger sequencing. Thus, DNA was isolated from EDTA blood of the ROSAH patient and an unrelated healthy volunteer using the DNeasy Blood & Tissue Kit (Quiagen) following the manufacturer's protocol. Polymerase chain Reaction (PCR) was subsequently performed on a T3000 Thermocycler (Biometra) using a Q5® High-Fidelity DNA Polymerase (New England BioLabs), Q5® Reaction Buffer (New England BioLabs). The following primers from Williams

et al. (2) were used for PCR reactions: GGCAAATAGTTCATATGGAGGAA (forward primers) and CCTCCCAATCTCCTGAGAAA (reverse primers).

Sample preparation for mass cytometry

Freshly isolated PBMCs were cultured for 4 h in RPMI 1640 supplemented with 10% fetal bovine serum, 1% Penicillin/Streptavidin and 1 µL/mL beta-mercaptoethanol (Sigma-Aldrich). Cells were either left unstimulated or subsequently treated for 4 h with either 20 ng/ml phorbol 12-myristate 13-acetate (PMA; Sigma-Aldrich) and 1 µg/ml ionomycin (Sigma-Aldrich) or with 100 ng/ml lipopolysaccharide (Sigma-Aldrich). To all conditions 5 µg/ml Brefeldin A (Sigma-Aldrich) was added. For the last 15 min of stimulations, cells were supplemented with 25 units/ml Benzonase (Sigma-Aldrich). Cells were fixed and frozen in Smart tube buffer (SMART TUBE inc.) and stored at -80°C before further analyses.

Barcoding and staining for mass cytometry

Fixed PBMCs were thawed at 37°C, washed with Maxpar Cell Staining Buffer (Fluidigm) and incubated with 25 units/mL Benzonase for 20 min at 37°C. Samples were then barcoded with six different palladium isotopes using the Cell-ID 20-Plex Pd Barcoding Kit (Fluidigm) following the manufacturer's protocol. After barcoding, samples were washed twice with Maxpar Cell Staining Buffer (Fluidigm) and pooled afterwards. Pooled cells were washed again with Maxpar Cell Staining Buffer (Fluidigm), and subsequently incubated with the antibody mix for cell surface staining (**Suppl. Table 1**) for 30 min at 4°C. After incubation, cells were washed twice with Maxpar Cell Staining Buffer (Fluidigm) and then incubated with fixation/permeabilization buffer (eBioscience) for 60 min at 4°C following the manufacturer's protocol. Cells were washed twice with permeabilization buffer (eBioscience) and subsequently incubated with the antibody mix for intracellular staining (**Suppl. Table 1**) for 60 min at room temperature (RT). Cells were then washed twice with permeabilization buffer (eBioscience) and twice with Maxpar Cell Staining Buffer (Fluidigm). Cells were subsequently incubated overnight in 2% methanol-free formaldehyde solution (ThermoFisher). After fixation, cells were washed twice with Maxpar Cell Staining Buffer (Fluidigm) and were incubated in iridium intercalator solution (Fluidigm) for 60 min at RT. Cells were then washed twice with Maxpar Cell Staining Buffer (Fluidigm) and washed with ddH2O using the Laminar Wash Mini (Curiox). Cells were kept at 4°C until CyTOF measurement.

CyTOF measurement and data analysis

Cells were acquired on a CyTOF2 mass cytometer upgraded to Helios specifications (CyTOF2/Helios) (Fluidigm). The instrument was tuned according to the manufacturer's instructions. EQ four element calibration beads (Fluidigm) were added to the sample for

normalization of signal changes over the time of the measurement. Data analysis was performed as previously described by Böttcher *et al.* (3). First, resulting flow cytometry standard (FCS) files were normalized and then uploaded to Cytobank (www.cytobank.org) for gating of single, live cells and de-barcoding. Individual FCS files were compensated using the R package CATALYST. Compensated files were again uploaded to Cytobank and reduced-dimensional (2D) t-SNE maps were generated. FCS files harboring the t-SNE data were downloaded from Cytobank und further analyzed using the R software. For cluster identification, FlowSOM/ConsensusClusterPlus was used.

Cultivation of *Helicobacter hepaticus*

Glycerol-stocked *Helicobacter hepaticus* (DSMZ, DSMZ no.: 22909) was grown in tryptone soya broth (TSB) supplemented with 10% FCS, 10 µg/mL Vancomycin, 5 µg/mL Trimethoprim und 2.5 IU/mL Polymyxin B (all from Sigma-Aldrich) in microaerophilic conditions. For stimulation of macrophages, *Helicobacter hepaticus* was harvested and washed twice with PBS.

Macrophage differentiation and stimulation

Monocytes were magnetically sorted from freshly isolated PBMCs using CD14 MicroBeads (Miltenyi) following the manufacturer's instructions. $CD14^+$ monocytes were cultured in RPMI 1640 supplemented with 10% fetal bovine serum and 1% Penicillin/Streptavidin for 7 days. For M1 polarization 5 ng/ml GM-CSF (Peprotech) was added to the culture, for M2 polarization cells were grown in the presence of 50 ng/ml M-CSF (Peprotech). M1 and M2 polarization was confirmed by microscopy and flow cytometry as described below. After 7 days of differentiation, macrophages were stimulated either with *Helicobacter hepaticus* (multiplicity of infection (MOI) of 100) or lipopolysaccharide (100 ng/ml; Sigma-Aldrich) for 4 h at 37°C. After incubation time, supernatant was collected and stored at -20°C until further analysis.

Confirmation of macrophage polarization by flow cytometry

Differentiation of monocytes into M1 and M2 macrophages was confirmed by flow cytometry. Thus, macrophages were harvested 6 days after start of differentiation by incubation with 5 mM EDTA in PBS for 10 min at 4°C. Harvested cells were washed once in MACS buffer (0.5% BSA in PBS) and incubated in the antibody mix for cell surface staining (**Suppl. Table 2**) for 20 min. Beriglobin was added to the antibody mix to block unspecific binding. Two min before the end of the incubation time, 0.1 µg/mL Dapi (Carl Roth) was added for live/dead staining. After incubation, cells were washed once with MACS buffer and samples were

measured with a Canto II flow cytometer (BD Bioscience). Data was analyzed with FlowJo software package V10.6 (FlowJo, LLC).

Flow cytometry analysis of T cells

Frozen Peripheral blood mononuclear cells (PBMCs) were thawed and subsequently stimulated as described in “Sample preparation for mass cytometry”. After stimulation, cells were washed once with PBS and then stained with 1:1000 Zombie Violet™ (Biolegend) for 10 min at 4°C to discriminate live and dead cells. Cells were washed once in MACS buffer (0.5% BSA in PBS) and then incubated in the antibody mix for cell surface staining (Suppl. Table 2) for 15 min at 4°C. After incubation, cells were washed once with PBS and then fixed in fixation buffer (eBioscience™ Foxp3/Transcription Factor Staining Buffer Set, Invitrogen) for 45 min at 4°C. Cells were washed once in permeabilization buffer (eBioscience™ Foxp3/Transcription Factor Staining Buffer Set, Invitrogen) and then incubated in the antibody mix for intracellular staining (Suppl. Table 2) for 25 at room temperature (RT). After incubation, cells were washed once with MACS buffer and samples were measured with a Canto II flow cytometer (BD Bioscience). Data was analyzed with FlowJo software package V10.6 (FlowJo, LLC).

Cytometric bead array

Concentrations of IL-6, TNFα and IL-10 in the supernatant of stimulated macrophages were determined with the human Inflammatory Cytokine Cytometric Bead Array (CBA) - I Kit (BD Biosciences) following the manufacturer’s protocol. Data was analyzed using the FCAP Array™ software V3.0 (BD Biosciences).

Microscopy

Pictures of macrophages at day 3 and day 6 of differentiation were taken using a Primovert microscope (Zeiss) at 20x magnification.

Graphs

All Graphs were generated using the Prism 9 software (GraphPad).

Supplementary Table 1: Antibodies for Mass Cytometry

Metall	Target	Clone/Company	Dilution
89Y	CD45	HI30/Fluidigm	1:100
142Nd	CD19	HIB19/Fluidigm	1:100
143Nd	CD45RA	HI100/Fluidigm	1:100
144Nd	IL-4	MP4-25D2/Fluidigm	1:100
145Nd	CD4	RPA-T4/Fluidigm	1:100

146Nd	TNFa	Mab11/Fluidigm	1:100
147Sm	CD11c	Bu15/Fluidigm	1:200
148Nd	IL17A	BL168/Fluidigm	1:100
149Sm	CD25	2A3/Fluidigm	1:100
150Nd	CD138	DL-101/Fluidigm	1:50
151Eu	CD103	Ber-ACT8/Fluidigm	1:50
152Sm	Fas	DX2/Fluidigm	1:50
153Eu	IgM	NHM-88/Biolegend	1:200
154Sm	CD3	UCTH1/Fluidigm	1:100
155Gd	CD56	Bd159/Fluidigm	1:50
156Gd	IL-6	MQ2-13AS/Fluidigm	1:50
158Gd	IFNg	B27/Fluidigm	1:200
159Tb	CCR7	G043H7/Fluidigm	1:200
160Gd	CD27	0323/Biolegend	1:200
161Dy	IL-23	23dcdp/Fluidigm	1:100
162Dy	CD8	RPA-T8/Fluidigm	1:100
163Dy	CD33	WM53/Fluidigm	1:100
164Dy	CD45RO	UCHL1/Fluidigm	1:100
165Ho	CD40	5C3/Fluidigm	1:50
166Er	IL-2	MQ117H12/Fluidigm	1:100
167Er	CD38	HIT2/Fluidigm	1:100
168Er	CD40L	24-31/Fluidigm	1:100
169Tm	IL-13	JES105A2/Fluidigm	1:50
170Er	IL-12	REA123/Miltenyi	1:50
172Yb	CX3CR1	2A9-1/Fluidigm	1:25
173Yb	HLA-DR	L243/Fluidigm	1:200
174Yb	PD-1	EH12.2H7/Fluidigm	1:200
175Lu	CD14	M5E2/Fluidigm	1:200
176Yb	IL-7R	A019D5/Fluidigm	1:50
209Bi	CD11b	ICRF44/Fluidigm	1:100

Supplementary Table 2: Antibodies for Flow Cytometry

**Target**	**Fluorochrome**	**Clone/Company**	**Dilution**
CD3	PE	HIT3a/Biolegend	1:100
CD4	BV510	RPA-T4/Biolegend	1:40
CD8	FITC	RPA-T8/Biolegend	1:100
CD14	APC	63D3/Biolegend	1:40
CD14	FITC	61D3/eBioscience	1:50
CD45RA	PE-Cy7	HI100/Biolegend	1:50
CD80	FITC	2D10.4/eBioscience	1:40
CD163	PE	GHI/63/BD	1:40
HLA-DR	PerCp-Cy5	LN3/eBioscience	1:100
IL-2	APC-Cy7	MQ1-17H127Biolegend	1:50
IFNγ	APC	4S.B3/Biolegend	1:40
TNFα	PerCP	MAb11/Biolegend	1:50

References

1. Ziegler JF, Bottcher C, Letizia M, Yerinde C, Wu H, Freise I, et al. Leptin induces TNFalpha-dependent inflammation in acquired generalized lipodystrophy and combined Crohn's disease. Nat Commun. 2019;10(1):5629.
2. Williams LB, Javed A, Sabri A, Morgan DJ, Huff CD, Grigg JR, et al. ALPK1 missense pathogenic variant in five families leads to ROSAH syndrome, an ocular multisystem autosomal dominant disorder. Genet Med. 2019;21(9):2103-15.
3. Bottcher C, Fernandez-Zapata C, Schlickeiser S, Kunkel D, Schulz AR, Mei HE, et al. Multi-parameter immune profiling of peripheral blood mononuclear cells by multiplexed single-cell mass cytometry in patients with early multiple sclerosis. Sci Rep. 2019;9(1):19471.

Supplementary Table 3: Treatment history of the ROSAH patient

Please note that the patient was admitted to our department for obtaining molecular diagnostics and a therapeutic recommendation. All medical information were retrieved from her medical record.

Treatment	Age [years]	Reason for discontinuation
Methotrexate	14-17	suspected neoplasia of the bladder/anemia
Sulfasalazine	17-35	inadequate effect
Leflunomide	35-35	inadequate effect
Adalimumab	35-36	recurrent infections
Etanercept	36-37	recurrent infections
Rituximab	37-45	secondary loss of effect
Baricitinib	45-46	inadequate effect
Tofacitinib	46-47	inadequate effect
Ustekinumab	47-47	inadequate effect
Adalimumab	47-	

Supplementary Table 4: Lymphocyte frequencies in the blood of the ROSAH patient

Reference ranges (14 years - 120 years): lymphocytes: 20-40%, $CD4^+$ T cells: 30-60%, $CD8^+$ T cells 20-40%

All medical information were retrieved from her medical record.

Age [years]	Lymphocytes [%]	$CD4^+$ T cells [%]	$CD8^+$ T cells [%]
14	reported leukopenia		
16	9,0	NA	NA
42	7,6	NA	NA
43	9,0	NA	NA
44	5,5	NA	NA
46	7,0	86,19	12,83

A

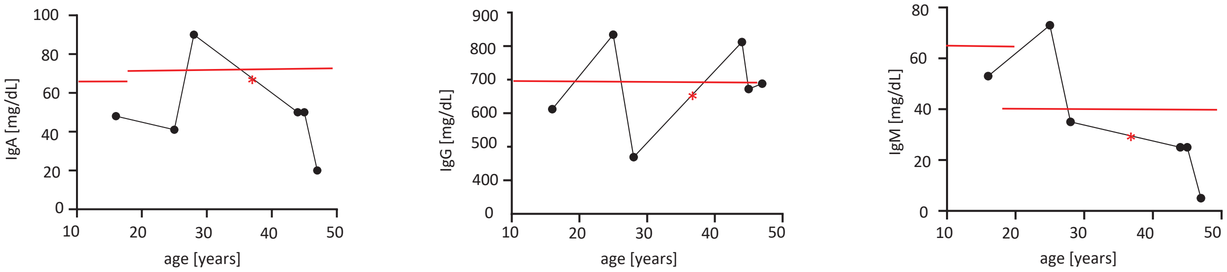

Supplementary Figure 1: Immunoglobulin levels in the serum of the ROSAH patient are reduced
A: Serum IgG, IgA and IgM concentration at different age of the ROSAH patient. Data was obtained from medical records of the patient and was acquired at various institutions. The red line indicates the 5th percentile for the respective immunoglobulin, the red star indicates start of Rituximab treatment.

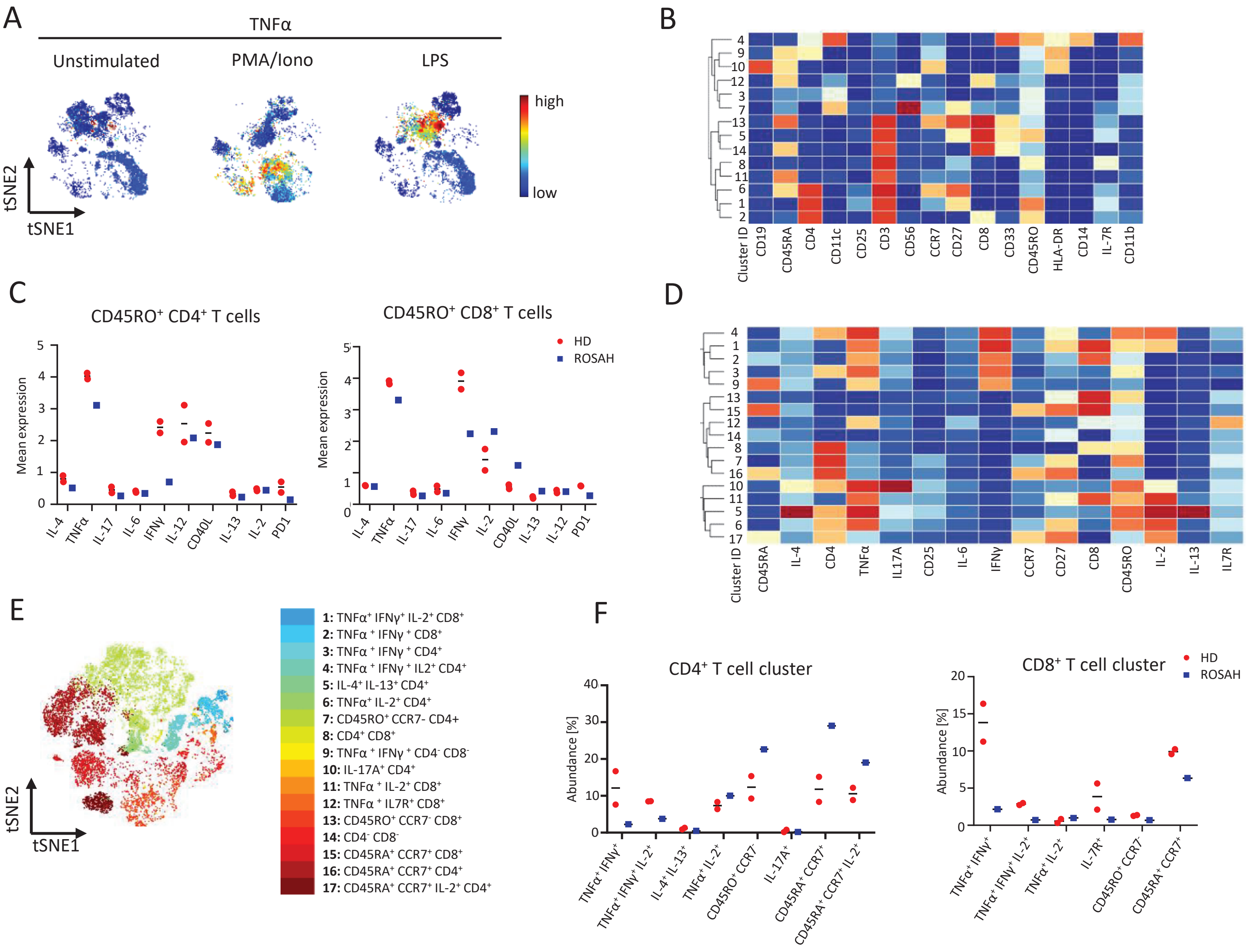

Supplementary Figure 2: Characterization of peripheral mononuclear cells of the ROSAH patient by mass cytometry

A-F: Peripheral blood mononuclear cells (PBMCs) of the ROSAH patient and healthy donors (HD) were stimulated with phorbol 12-myristate 13-acetate(PMA)/ ionomycin (Iono) or lipopolysaccharide (LPS) and analyzed by mass cytometry. **A)** Representative t-SNE plots show the effect of different stimulations on the expression of TNFα. **B:** Heat map showing the expression of 16 selected markers used for clustering of $CD45^+$ PBMCs of the ROSAH patient and HDs. **C:** Mean expression of selected markers in $CD45RO^+$ $CD4^+$ and $CD45RO^+$ $CD8^+$ cells of PMA/Iono stimulated PBMCs **D:** Heat map showing the expression of 15 selected markers used for clustering of $CD3^+$ cells. **E:** t-SNE plot of $CD3^+$ cells colored by the 17 identified clusters**. F:** Abundance of 8 identified clusters in $CD4^+$ cells and 6 identified clusters in $CD8^+$ cells of PMA/Iono stimulated PBMCs

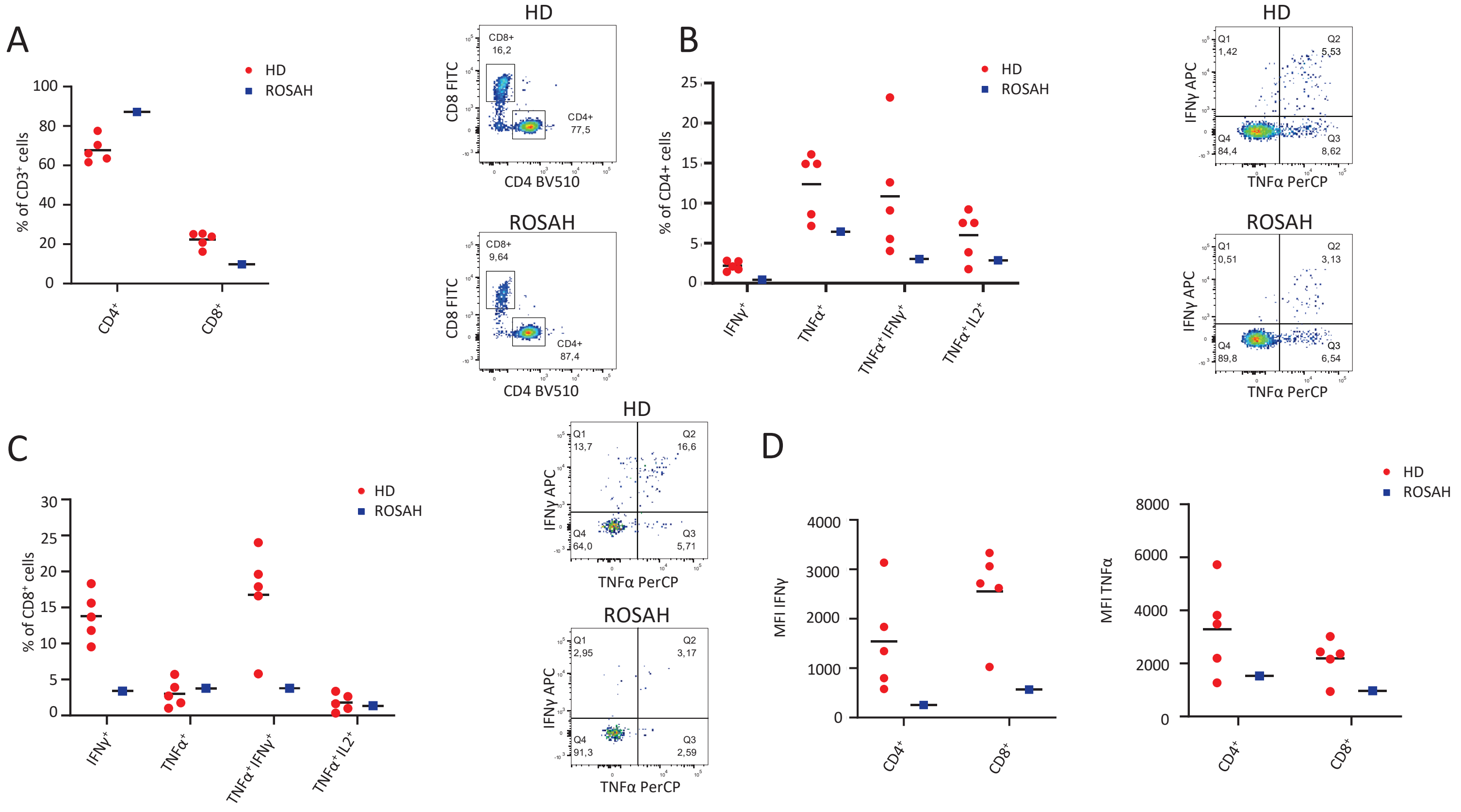

Supplementary Figure 3: IFNγ and TNFα expression is reduced in T cells of the ROSAH patient

A-D: Frozen peripheral blood mononuclear cells (PBMCs) of the ROSAH patient and healthy donors (HD) were thawed and subsequently stimulated with phorbol 12-myristate 13-acetate(PMA)/ionomycin (Iono) and analyzed by flow cytometry. **A:** Frequency of $CD4^+$ and $CD8^+$ T cells among $CD3^+$ T cells. Representative flow cytometry plots are depicted next to the graph. **B:** Frequency of $IFN\gamma^+$, $TNF\alpha^+$, $TNF\alpha^+$ $IFN\gamma^+$ and $TNF\alpha^+$ $IL\text{-}2^+$ cells among $CD4^+$ T cells. Representative flow cytometry plots are depicted next to the graph. **C:** Frequency of $IFN\gamma^+$, $TNF\alpha^+$, $TNF\alpha^+$ $IFN\gamma^+$ and $TNF\alpha^+$ $IL\text{-}2^+$ cells among $CD8^+$ T cells. Representative flow cytometry plots are depicted next to the graph. **D:** Mean fluorescence intensity (MFI) of TNFα and IFNγ in $CD4^+$ and $CD8^+$ T cells.

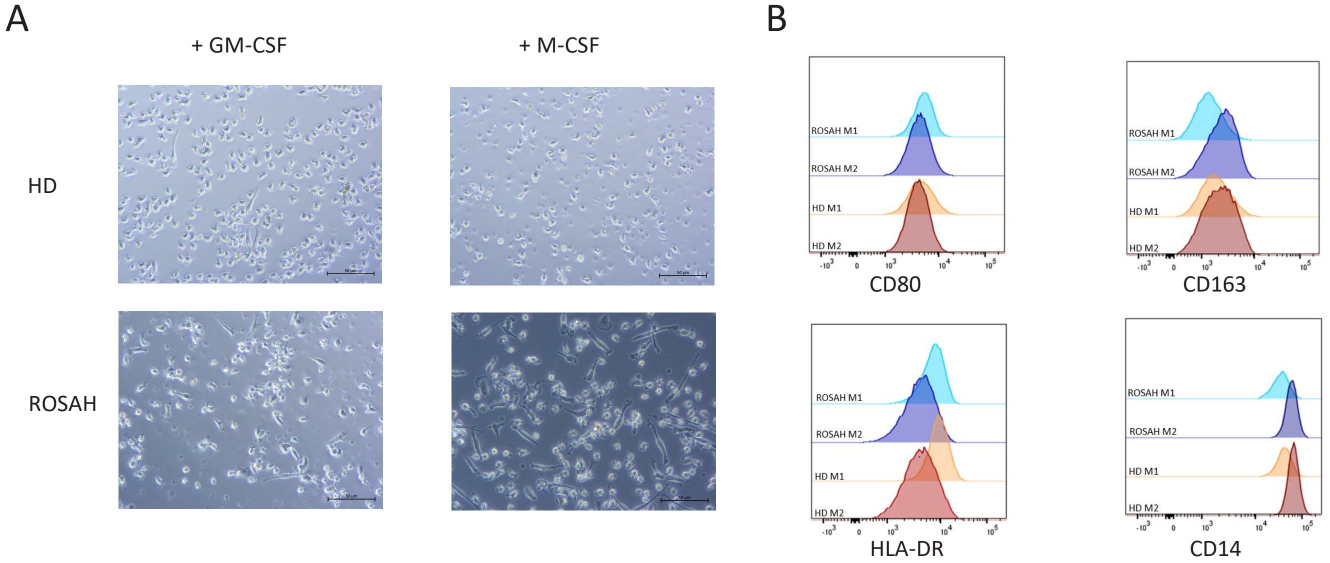

Supplementary figure 4: Comparable *in-vitro* differentiation of macrophages of the ROSAH patient and a healthy donor
A-B: Blood-derived monocytes of the ROSAH patient and one HD were differentiated into M1 and M2 macrophages by supplementation of GM-CSF or M-CSF for 7 days. **B:** Microscopic pictures of differentiated macrophages 6 days after start of polarization. Scale bar depicts 50 µm. **C:** Analysis of differentiated macrophages 6 days after start of polarization by flow cytometry.
